# A Maximum Entropy Model of the Distribution of Dengue Serotype in Mexico

**DOI:** 10.1155/2023/3823879

**Published:** 2023-07-25

**Authors:** Esther Annan, Jailos Lubinda, Jesús Treviño, William Messer, Dina Fonseca, Penghua Wang, Jurgen Pilz, Benjamin Lintner, Aracely Angulo-Molina, Ana L. Gallego-Hernández, Ubydul Haque

**Affiliations:** ^1^Center for Health and Wellbeing, School of Public and International Affairs, Princeton University, Princeton, NJ, USA; ^2^Malaria Atlas Project, Telethon Kids Institute, 6009, Nedlands, WA, Australia; ^3^Department of Urban Affairs at the School of Architecture, Universidad Autónoma de Nuevo León, San Nicolás de los Garza, Nuevo Léon 66455, Mexico; ^4^Department of Molecular Microbiology and Immunology, Oregon Health and Science University, 3181 SW Sam Jackson Park Road, Portland, OR 97239, USA; ^5^Department of Medicine, Division of Infectious Disease, Oregon Health and Science University, Portland, OR, USA; ^6^Center for Vector Biology, Rutgers University, New Brunswick, NJ, USA; ^7^Department of Immunology, School of Medicine, U Conn Health, Farmington, CT 06030, USA; ^8^Department of Statistics, University of Klagenfurt, Klagenfurt, Austria; ^9^Department of Environmental Sciences, Rutgers, The State University of New Jersey, New Brunswick, NJ, USA; ^10^Departamento de Ciencias Químico-Biológicas, Universidad of Sonora, Hermosillo 83000, Mexico; ^11^Rutgers Global Health Institute, New Brunswick, NJ, USA; ^12^Department of Biostatistics and Epidemiology, School of Public Health, Rutgers University, Piscataway, NJ, USA

## Abstract

Pathogen strain diversity is an important driver of the trajectory of epidemics. The role of bioclimatic factors on the spatial distribution of dengue virus (DENV) serotypes has, however, not been previously studied. Hence, we developed municipality-scale environmental suitability maps for the four dengue virus serotypes using maximum entropy modeling. We fit climatic variables to municipality presence records from 2012 to 2020 in Mexico. Bioclimatic variables were explored for their environmental suitability to different DENV serotypes, and the different distributions were visualized using three cutoff probabilities representing 90%, 95%, and 99% sensitivity. Municipality-level results were then mapped in ArcGIS. The overall accuracy for the predictive models was 0.69, 0.68, 0.75, and 0.72 for DENV-1, DENV-2, DENV-3, and DENV-4, respectively. Important predictors of all DENV serotypes were the growing degree days for December, January, and February, which are an indicator of higher temperatures and the precipitation of the wettest month. The minimum temperature of the coldest month between −5°C and 20°C was found to be suitable for DENV-1 and DENV-2 serotypes. Respectively, above 700–900 mm of rainfall, the suitability for DENV-1 and DENV-2 begins to decline, while higher humidity still favors DENV-3 and DENV-4. The sensitivity concerning the suitability map was developed for Mexico. DENV-1, DENV-2, DENV-3, and DENV-4 serotypes will be found more commonly in the municipalities classified as suitable based on their respective sensitivity of 91%, 90%, 89%, and 85% in Mexico. As the microclimates continue to change, specific bioclimatic indices may be used to monitor potential changes in DENV serotype distribution. The suitability for DENV-1 and DENV-2 is expected to increase in areas with lower minimum temperature ranges, while DENV-3 and DENV-4 will likely increase in areas that experience higher humidity. Ongoing surveillance of municipalities with predicted suitability of 89% and 85% should be expanded to account for the accurate DENV serotype prevalence and association between bioclimatic parameters.

## 1. Introduction

The distribution of *Aedes aegypti* and dengue virus (DENV), the cause of dengue fever (DF), is expanding rapidly and globally in tropical megacities, including those in Mexico [1, 2]. *A*. *aegypti*, the primary vector of DF, is spatially confined mostly in tropical and subtropical climates with some limited occurrences in temperate climates [3]. Within these regions, the distribution of DF-specific DENV serotypes is, however, not uniform. This occurs partly as a result of human host background immunity [4, 5] and virus genetic diversification [6], existing weather patterns, and factors like climate change [7]. Dengue virus serotype-1 (DENV-1) was first detected in the America in 1977, followed by DENV-2, DENV-4 in 1981, and then DENV-3 in 1994 [6]. Between 1981 and 1997, severe DF, which can be fatal, was reported in 24 countries in the America, including Mexico [8, 9]. The first occurrence of severe DF reported in Mexico was in 1985 [6, 10]. Since then, there has been an introduction and redistribution of different serotypes across states in Mexico [6, 11–15].

Currently, there is only one approved vaccine in Mexico, known as CYD-TDV or “Dengvaxia” by Sanofi Pasteur, and its efficacy is dependent on the DENV serotype, baseline serostatus, and age [16, 17] of vaccinees. Some studies have linked Dengvaxia-elicited responses to low antibody titers against DENV-2 in certain populations [18, 19]. However, a recent study has suggested that a tetravalent dengue vaccine (TAK-003) by Takeda vaccines may be well tolerated in unexposed adolescents but had limited efficacy against DENV-3 [20, 21]. The choice of dengue vaccine or dengue prevention may, therefore, benefit from knowledge about DENV serotype presence and predicted pattern of spread.

The co-circulation of different dengue virus (DENV) serotypes and the presence of more virulent variants in the general population have been associated with outbreaks in some regions at risk of DF [22, 23]. Southeast Asian DENV-3 has been associated with severe dengue among individuals with primary infection, while non-Southeast Asian DENV-2 and DENV-3 have been found to increase the risk of severe dengue among those with secondary infection [24]. This suggests a possible correlation between DENV-serotype, its origin, and DF severity.

In this study, we modeled the environmental suitability of the four DENV serotypes to predict their potential geographic ranges and to compare these ranges with current trends observed across Mexico. We hypothesized that the distribution of DENV serotypes would vary across Mexico and that the differences in distribution would be influenced by environmental factors. Identification of climate-sensitive risk factors to predict serotype distribution at the municipality level will provide indices that may help in outbreak preparedness. Furthermore, the pattern of serotype distribution may also assist in the planning for dengue vaccination across Mexico.

## 2. Methods

### 2.1. Serotype Data

Serotype data were retrieved from Mexico's Ministry of Health (MoH) for analysis. This dataset contains nonidentifiable health information, including laboratory-confirmed serotypes from 2,469 Mexican municipalities collected from notifying units between 2012 and 2020. DENV serotype was determined using quantitative reverse transcription polymerase chain reaction (PCR), which is the main diagnostic for DENV serotypes in Mexico [25]. A serotype was classified as being present in a municipality if there were one or more records of a positive PCR result between 2012 and 2020.

### 2.2. Preparing the Bioclimatic Data

We used the daily temperature values to compute the daily growing degree days (GDD), during December, January, and February (GDD), a measure of the magnitude by which daily average temperatures exceed a baseline temperature of 10°C. Cumulative GDDs during each quarter of the year were then computed as a measure of total warmth during winter. GDD was calculated from precipitation and temperature data obtained from 1993 to 2016 by NASA and compiled with Daymet version 3 [26]. The temperature and precipitation data were obtained with a 1 km *×* 1 km spatial resolution for compilation by Daymet [26]. Daymet is a research product that provides gridded estimates of climatic variables through interpolation and extrapolation. Daily fields such as minimum temperature, maximum temperature, and precipitation were used to calculate the GDD and 19 bioclimatic variables of interest in ecological modeling (Table 1). The “dismo” package version 1.1-1 was used to calculate the bioclimatic variables in R [27].

### 2.3. Determining the Environmental Suitability Model

Bioclimatic variables were imported into ArcGIS [28] and projected (Mexico ITRF92/UTMzone 16 N) as a raster image in ArcGIS 10.3 for prediction mapping. A correlation test was then performed with all bioclimatic variables to test for multicollinearity and to exclude variables that were highly correlated (Pearson *r* > 0.80).

The correlation test was performed in ArcGIS, using the multivariate band collection statistics tool. In ArcGIS, a shapefile was created to connect DENV serotype presence information with the centroids of their respective municipalities using municipality ID codes. This was later converted into ASCIan I file. Coordinates of municipalities where DENV-1, DENV-2, DENV-3, and DENV-4 were present (presence points) were then projected in ArcGIS.

### 2.4. Maxent Modeling Program

Maximum entropy (Maxent) is a program used for modeling species by using presence only [29]. Species distribution modeling is used to determine the relationship between the records of species and the spatial characteristics of their environment [29]. Non-random relationships are detected between geocoded locations of species and raster images that are representative of the environment to determine the propensity of a species to occur in that study area [30]. In this study, Maxent models were used to determine the probability of the presence of DENV-1, DENV-2, DENV-3, and DENV-4 serotypes independently (*y* = 1) and this was conditional on environmental covariates (*z*). The equation (1) is given below.

(1) $$\begin{array}{c} Pr\left(y=1\left|z\right.\right)=f1\left(z\right)Pr\left(y=1\right)/f\left(z\right), \end{array}$$

where *f*_1_(*z*) is the probability density of environmental covariates in locations where the DENV serotype is present, *f* (*z*) is the probability density of the covariates across the locations, and Pr (*y* = 1) is the prevalence of the DENV serotype [29]. To approximate the prevalence, Maxent outputs a logistic function *ƞ* (z) = log (*f*_1_(*x*)/*f*(*z*)), while calibrating the intercept so that the average value of *ƞ* (*z*) is a parameter which represents the prevalence [29].

From 2012 to 2020, laboratory-confirmed 71,058 serotypes (DENV-1 [*N* = 33,794 (47%)], DENV-2 [*N* = 35,793 (50%)], DENV-3 [*N* = 950 (1.34%)], and DENV-4 [*N* = 521 (0.73%)]) were detected in Mexico. There were 1,519 samples of serotype-presence points (DENV-1, DENV-2, DENV-3, and DENV-4) that were prepared for analysis at the municipality level (e.g., presence/absence of DENV-1 in each municipality: yes/no). As previously done [31], for every municipality where each individual serotype was recorded, two municipalities that were lacking serotype records were randomly selected as background points. This was achieved by multiplying the presence points for each serotype by two with the aim of selecting background data with the same bias and, therefore, addressing potential selection bias [32, 33]. Hence, background points for DENV-1, DENV-2, DENV-3, and DENV-4 were 1,428, 1,290, 222, and 98, respectively. Twenty bioclimatic variables were tested for correlation. Sixteen of them had a Pearson correlation (*r*) < 0.8 and were retained. Each model was replicated 10 times for each serotype, with 10-fold cross-validation, using points in the model building and evaluation process.

To examine each bioclimatic variable's predictive potential of influencing serotype distribution, the permutation of importance, jackknife plots, and response curves were used as diagnostic criteria [31]. For each model, the area under the curve (AUC) of the receiver operating characteristic (ROC) curve was used to assess model fit and accuracy. Response curves were generated to show how each bioclimatic factor affected the model prediction while keeping other variables constant at their sample means. Variables that contributed less than 5% to the model were then removed from the model.  Table 2 shows the predictor variables that were retained in the model for each serotype.

### 2.5. Sensitivity Analysis

Varying cutoff probabilities, representing 91%, 90%, 89%, and 85% sensitivity for DENV-1, DENV-2, DENV-3, and DENV-4, respectively, were used to visualize the serotype distribution models in ArcGIS 10.7.1

### 2.6. Coding and Environment

Data preprocessing, statistical analysis, and generation of results were done using ArcGIS Desktop (version 10.7.1 ESRI Inc.) and maximum entropy species distribution modeling (version 3.4.4).

## 3. Results

**Figure 1:**
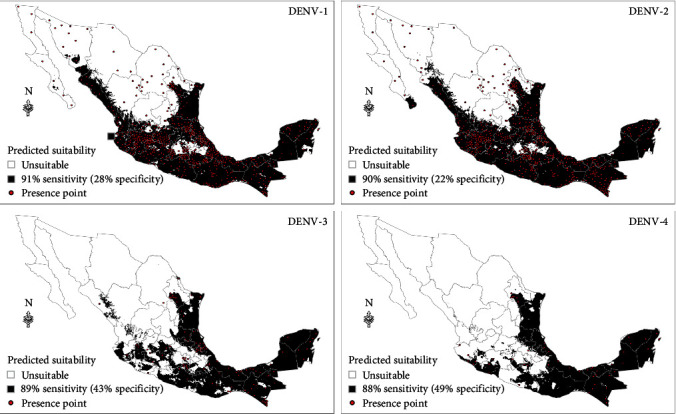
Modeled suitability maps of DENV-1, DENV-2, DENV-3, and DENV-4 at a sensitivity of 91%, 90%, 89%, and 88%, respectively. Red points show presence records from 2012 to 2020 used to build the models.

Results show statistically significant overlaps in modeled suitability for all four DENV species across Mexico. Compared to DENV-1 and DENV-2, DENV-3 and DENV-4 have smaller ranges of suitability extending to Southeastern Mexico, along the Gulf of Mexico (Figure 1). In contrast, DENV-1 and DENV-2 suitability extend to parts of northwestern Mexico. Furthermore, sporadic distributions of transmission of DENV-1 and DENV-2 serotypes were also present in predicted unsuitable regions of Mexico. While regions of overlap and presence of dengue in unsuitable regions occur in the southern and northern parts of Mexico, respectively, there are areas in central Mexico that were unsuitable for all DENV serotypes.

The mean AUC for DENV-1, DENV-2, DENV-3, and DENV-4 was 0.69, 0.68, 0.75, and 0.72, respectively (Table 1). The GDD was the most important bioclimatic predictor of DENV-1, DENV-2, and DENV-3 suitability, while the mean diurnal range was the most important predictor for DENV-4 suitability (Table 1). Precipitation of the wettest month was also an important predictor for DENV-2, DENV-3, and DENV-4 but less important for DENV-1 compared to the other three serotypes. While temperature generally predicted the DENV serotype distributions to a large extent, specific measures associated with variance in temperature differed in importance when determining serotype distribution. While colder temperatures were associated with DENV-1 and DENV-2, higher precipitation ranges were associated with DENV-3 and DENV-4. The mean diurnal range had a relatively higher permutation of importance for DENV-3 and DENV-4 compared to DENV-1 and was excluded from the DENV-2 model due to a less than 5% permutation importance. The minimum temperature of the coldest month had relatively high permutations of importance for DENV-1 and DENV-2 but not for DENV-3 and DENV-4.

**Figure 2:**
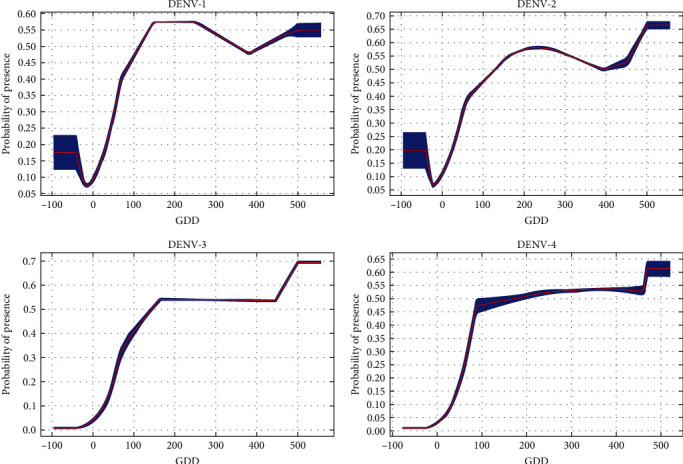
Model response curves for DENV-1, DENV-2, DENV-3, and DENV-4 suitability about growing degree days for December, January, and February.

**Figure 3:**
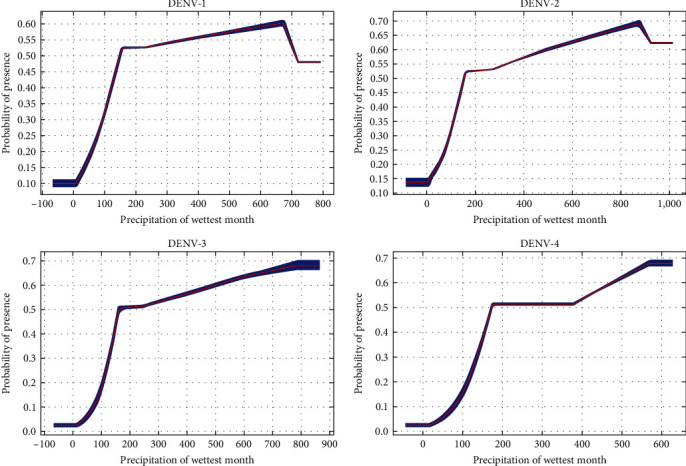
Model response curves for DENV-1, DENV-2, DENV-3, and DENV-4 suitability in relation to precipitation of wettest month.

**Figure 4:**
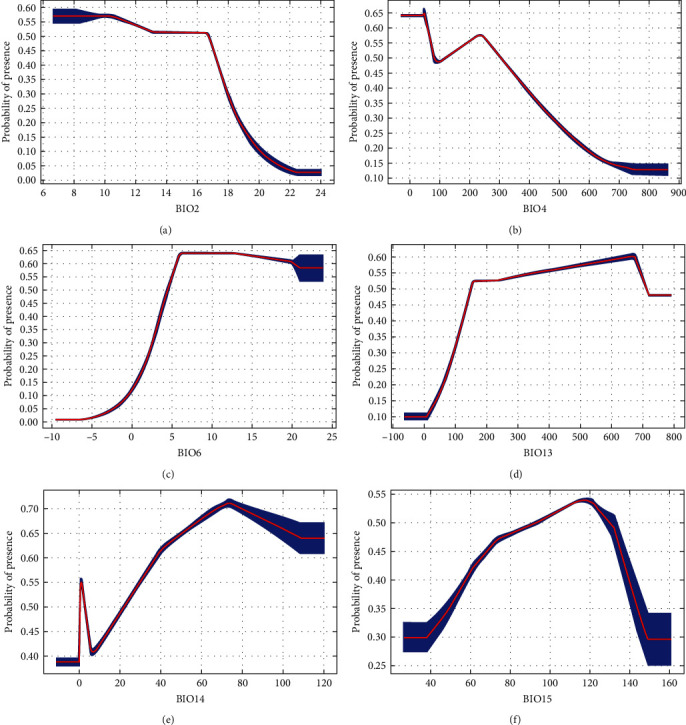
Model response curves for DENV-1 about (a) mean diurnal range (monthly (maximum temperature–minimum temperature)), (b) temperature seasonality, (c) minimum temperature of coldest month, (d) precipitation of wettest month, (e) precipitation of driest month, and (f) precipitation seasonality (coefficient of variation).

**Figure 5:**
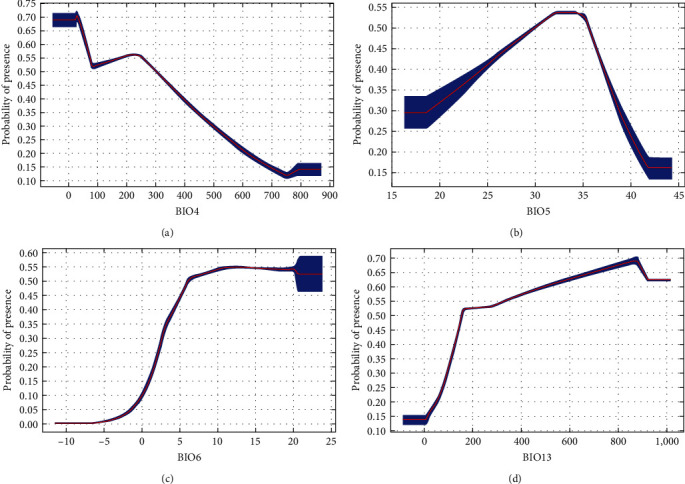
Model response curves for DENV-2 about (a) temperature seasonality, (b) maximum temperature of warmest month, (c) minimum temperature of coldest month, and (d) precipitation of the wettest month.

**Figure 6:**
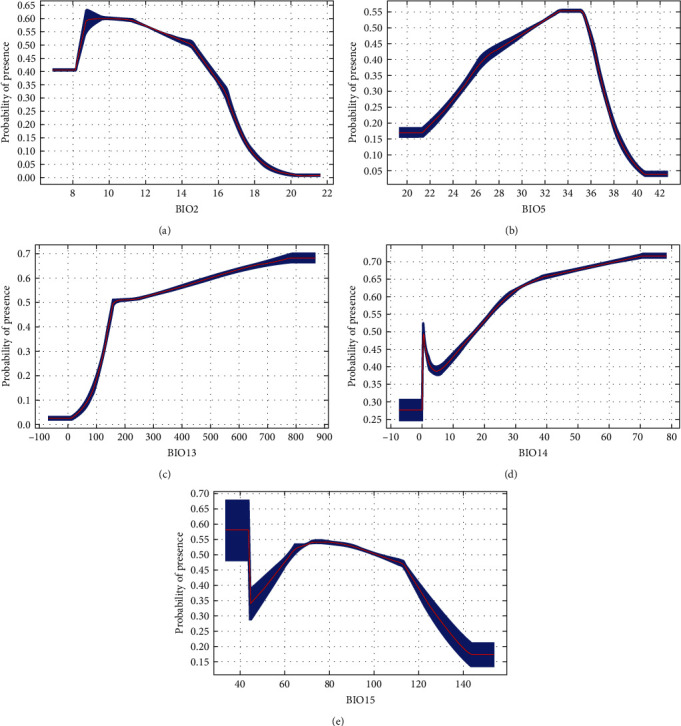
Model response curves for DENV-3 about (a) mean diurnal range (monthly (maximum temperature–minimum temperature)), (b) maximum temperature of warmest month, (c) precipitation of wettest month, (d) precipitation of driest month, and (e) precipitation seasonality (coefficient of variation).

**Figure 7:**
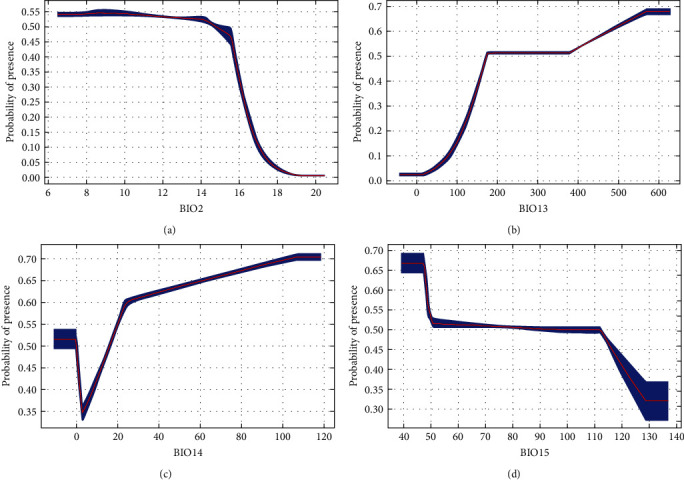
Model response curves for DENV-4 about (a) mean diurnal range (monthly (maximum temperature–minimum temperature)), (b) precipitation of wettest month, (c) precipitation of driest month, and (d) precipitation seasonality (coefficient of variation).

If average temperatures exceed 10°C in winter, the municipality would be suitable for all DENV serotypes (Figure 2). There was a steeper rise for DENV-1 from 0 to 100. Between 200 and 400, there were fluctuations for both DENV-1 and DENV-2. However, for DENV-3 and DENV-4, there remained about a 50% probability of presence and then, there is suitability for all serotypes above 400. Above 100 mm of rainfall, suitability for DENV-1 and DENV-2 increases until it starts dropping at higher rainfall levels (above 700 and 900 mm, respectively) (Figure 3). On the contrary, there is an upward trend for DENV-3 and DENV-4, particularly for DENV-3 beyond 800 mm, where suitability remains around 70%. Suitability increased for DENV-1 and DENV-2, with minimum temperature of the coldest month from -5°C to 20°C (Figures 4 and 5). Suitability for DENV-3 increased between a mean diurnal range of 8°C and 9°C, after which it decreased. On the contrary, suitability for DENV-4 is high between 6°C and 14°C, after which it decreases (Figures 6 and 7). Winter (December, January, and February) GDD higher than 0 was associated with moderate suitability.

## 4. Discussion

Based on our model, there is an overall suitability of all four DENV serotypes in southeastern Mexico, and there is a presence of DENV-1 and DENV-2 in both suitable and unsuitable regions of the central west and northern Mexico. Intervention strategies targeting southeastern Mexico may, therefore, help to hamper the distribution and spread of all four DENV serotypes.

Sporadic distributions of the DENV serotypes to currently unsuitable regions could point to environmental factors and/or an increase in human movement, which aids in the transport of mosquitoes to cities where the species survive. Species distribution models have predicted that new areas around current inhabitable geographic regions of the *Aedes* spp. will become environmentally suitable for the mosquito's life cycle and transmission in urban areas [34]. This supports the observation of DENV-1 and DENV-2 in unsuitable areas, particularly in areas adjacent to suitable regions. Variability in reporting across regions may also account for the variations seen across municipalities. However, this is unlikely the case, as DF is endemic in Mexico with increased availability of rapid diagnostic tests since the 1990s [35]. On a molecular level, serotypes with specific genotypic advantages may outperform other serotypes [36]. It could be possible that a microclimatic parameter influences one serotype, its transmission, vectoral adaptivity, or infectivity in humans, etc. Mosquitoes are cold-blooded and their body temperatures may fluctuate with ambient temperatures, which may influence viral replication in mosquitoes. Certain serotypes or genotypes may tolerate temperature fluctuation and replicate better than others [37].

Our study supports other findings [38] that also strongly suggest distinct ecological preferences by DENV larvae based on their serotypes. This may suggest *Aedes* larvae sensitivity to water temperature, turbidness, and other conditions affecting larvae survival in pools, as such partly explaining DENV serotype-specific preferences in relation to habitats [39–42].

More broadly, studies have shown that bioclimatic variables have significant influence on dengue virus transmission and distribution by affecting the characteristics of the mosquito population and the suitability of their habitats. These directly or indirectly affect the mosquito habitats and the characteristics of the mosquito population, including virulence. Recent studies [43, 44] reveal that temperature and rainfall accelerate the extrinsic incubation period of the virus in the mosquito and increase the mosquito's density and biting rate, which could result in increased transmission of the dengue virus. These studies all observe and suggest potential serotype associations with environmental, ecological, and broad bioclimatic covariates.

Another study [38] reported periodic shifts in the predominant DENV serotypes, exhibiting a simultaneous circulation of all four DENV serotypes identified in their study area. This study broadly suggests observable associations between serotypes and microchanges in bioclimatic variables that could be seasonally driven, thereby driving periodic serotype shifts.

The climatic zone is a vital factor that substantially affects the spread of dengue infection [45]. The literature also states that the bioclimatic zone greatly affects the transmission of dengue virus serotypes [46]. The rationality of this argument is that rainfall plays a critical role since water is needed to hatch mosquito eggs and develop the larval stage. *Aedes* mosquitoes breed mainly in various containers with stagnant water like plastic buckets, puddles, or coconut shells. It is statistically tested that dengue risk increases after 6–10 weeks after rainfalls above 300 mm per week and a 4-week lag of increasing temperature [46].

The geographic distribution of dengue virus serotypes can vary spatially and temporally due to a variety of factors, including the level of infection spread by the mosquitoes, climate variation, circulation of different serotypes, and the population's immunological history [47, 48]. Other factors that can influence the spatial distribution of virus serotypes include the spatial distribution and movement of mosquitoes and humans, life span, and socioeconomic factors [5]. Lower socioeconomic status, possibly due to crowding, poor hygiene, or open space economic activities, increases the opportunities for transmission [5]. Other socioeconomic factors that may account for these variances include the amount of travel, migration, and engagement in commercial trade across regions [35]. Fumigation campaigns to eradicate mosquitos might also impact the serotype-distribution since campaigns usually depend on municipality rules.

The limitation of DENV-3 and DENV-4 in the southeastern region might be due to how DENV serotypes migrate across Mexico. Historically, the southern states in Mexico have been the first and most affected by DF [49]. Furthermore, it has been hypothesized that there is a periodic introduction of dengue from Central America through the southern frontier [49, 50]. Hence, the spatially concentrated presence of DENV-1 and DENV-2, which are older serotypes in Mexico [49], in both southern and northern regions may be a reflection of human migration patterns. The absence of DENV serotypes and the unsuitability of regions in central Mexico for *A. aegypti* may be attributable to the high altitude of the region. The central region of Mexico has higher altitude ranges compared to the other regions and prior literature suggests a reduced presence of *A. aegypti* above 1,700 m [51]. It has also been argued that the absence of a species in an environmentally unsuitable region may be a result of local extinctions due to factors like dispersal limitations, biotic interactions, and previous environmental disturbances like fires, landslides, and flooding [29].

Prior studies [2, 34, 52] have linked increased precipitation and locally suitable temperatures to an increased risk of DF. Particularly, the presence of *A*. *aegypti* has been shown to have a positive association with the minimum temperature of the coldest season, mean precipitation of the wettest month, and GDD [53]. Peak DF cases have been associated with maximum temperatures of about 24–35°C, while large diurnal temperature ranges at lower temperatures tend to result in an increase in the number of dengue cases [54, 55]. From our study, strain-specific differences are seen, with lower temperatures favoring DENV-1 and DENV-2, a narrow mean diurnal range of 8–9°C favoring DENV-3, and a wider mean diurnal range of 6–14°C favoring DENV-4. Published studies [56] suggest that at a lower temperature (i.e., 8°C), larvae die within a couple of days. The predicted temperatures also remain within baseline thresholds as precited by other studies [57]. The effect of the environment on viral accumulation, virulence, and adaptation [58] may explain the serotype-specific differences seen within the *A*. *aegypti* species. In addition to local temperatures, humidity, vapor pressure, and proximity to low-income and periurban regions have also been found to be associated with DF in some locations [59, 60]. Higher precipitation indices were common for both DENV-3 and DENV-4. Further, the abundance of one serotype compared to the other three serotypes in a region may be due to a competitive advantage of one serotype over the other [61].

This study has several limitations. With presence-only data, there is an inability to directly determine the prevalence of DENV serotypes, i.e., the proportion of the serotypes in the occupied site to the entire landscape. In the absence of the parameter *ƞ*(*z*), which is used to estimate the prevalence, Maxent sets an arbitrary prevalence of 0.5. Another limitation that is considered fundamental to presence-only data is sample selection bias [29], where a specific covariate space may be mapped out, giving an estimate that reflects the presence of a species concerning that covariate. To address potential selection bias, we used background data that had the same bias (two times the presence points) as the species occurrence data, as suggested in previous studies [32, 33]. Finally, future studies will need to be conducted to understand how genetic differences between DENV serotypes can explain how landscape-level rainfall and temperature ranges can impact DENV distribution in a serotype-specific manner.

## 5. Conclusion

Climate influences the variability of *A*. *aegypti* distribution, and this effect is further varied for DENV-specific serotypes. The GDD was the most important predictor of DENV-1, DENV-2, and DENV-3, while the mean diurnal range predicted the DENV-4 distribution best. Our study suggests that in the coldest month, lower temperatures may favor DENV-1 and DENV-2 suitability, while higher humidity ranges may favor DENV-3 and DENV-4 suitability. These findings may help to explain and predict patterns of DENV-serotype distributions that may emerge in the future based on climate change. Furthermore, monitoring of DENV-specific serotypes may be guided by using the best predictive bioclimatic factors associated with each serotype.

## Figures and Tables

**Table 1:** Bioclimatic variables.

Full meaning	Bioclimatic variable
Annual mean temperature	BIO1
Mean diurnal range (monthly (maximum temperature–minimum temperature))	BIO2
Isothermality (mean diurnal range/temperature annual range) × 100	BIO3
Temperature seasonality	BIO4
Maximum temperature of warmest month	BIO5
Minimum temperature of coldest month	BIO6
Temperature annual range	BIO7
Mean temperature of wettest quarter	BIO8
Mean temperature of driest quarter	BIO9
Mean temperature of warmest quarter	BIO10
Mean temperature of coldest quarter	BIO11
Annual precipitation	BIO12
Precipitation of wettest month	BIO13
Precipitation of driest month	BIO14
Precipitation seasonality (coefficient of variation)	BIO15
^*∗*^Precipitation of wettest quarter	BIO16
^*∗*^Precipitation of driest quarter	BIO17
^*∗*^Precipitation of warmest quarter	BIO18
^*∗*^Precipitation of coldest quarter	BIO19
Growing degree days for December, January, and February	GDD

^*∗*^Highly correlated bioclimatic variables with Pearson correlation (*r*) > 0.8.

**Table 2:** Predictor variables used in the environmental suitability models for DENV-1, DENV-2, DENV-3, and DENV-4.

	Permutation importance (%) ^*∗*^			
Variable	DENV-1	DENV-2	DENV-3	DENV-4
Mean diurnal range (mean of monthly (maximum temperature–minimum temperature))	6.8	–	17.8	35.8
Temperature seasonality (standard deviation × 100)	8.5	8.9	–	–
Maximum temperature of warmest month	–	8.4	9.3	–
Minimum temperature of coldest month	14.1	21.6	–	–
Precipitation of wettest month	9.1	22	25.6	21.4
Precipitation of driest month	7.8	–	5.9	7.9
Precipitation seasonality (coefficient of variation)	12.9	–	7.1	12.8
Growing degrees days for December, January, and February	40.8	39.1	34.4	22.1

^*∗*^The percentages of “permutation of importance” are given for variables that were retained in the final models.

## Data Availability

The data that support the findings of this study will be made available in the supplementary material of this article.
